# Post-Mortem Examination of a Soldier One Hundred and Two Years Old

**Published:** 1842-11

**Authors:** 


*Post-Mortem Examination of a Soldier one hundred and two years old.*—This man had taken part in numerous campaigns, and had never suffered from illness. He retained his bodily powers up to his hundredth year, but gave up from decrepitude and mental incapacity fifteen months previously to his death, and was received into the Military Hospital of Bradenburgh. His death occurred unexpectedly and without premonitory indication.

On examination, thirty-six hours after decease, the body was found moderately stout; the few hairs that remained were silvery; his teeth had long since quitted their sockets, the alveolar processes of the jaws were completely absorbed, and the mouth contracted in size. The opening of the cranium was rendered difficult by the thickness of the bones and their adhesion with the dura mater, and the latter was dense and tendinous. The brain was normal, its arteries converted into bony tubes; the pineal gland was filled with calcareous matter, and the choroid plexuses were vesicular. The larynx and carotida were completely ossified. The thorax large and well formed; lungs perfectly sound; heart normal; valves remarkably ossified; large arteries converted into ossific tubes; costal cartilages almost completely ossified. The liver, spleen, and kidneys presented a state of extreme softening, greater than could be referred to decomposition, and which can alone be explained on the supposition of excessive debility of the vital powers.

Thus the kidneys consisted of a semifluid granular mass, although the urine was natural and unclouded. The gall-bladder was distended with upwards of five hundred concretions, of various magnitude, and its coats were so soft that they became ruptured by the slightest touch. No symptoms of this collection had existed during life.—*London Lancet*, Aug. 13, 1842, from *Medicinische Zeitung*, 1842.

